# NSD1 Mitigates Caspase-1 Activation by Listeriolysin O in Macrophages

**DOI:** 10.1371/journal.pone.0075911

**Published:** 2013-09-18

**Authors:** Olivia S. Sakhon, Kaitlin A. Victor, Anthony Choy, Tokuji Tsuchiya, Thomas Eulgem, Joao H. F. Pedra

**Affiliations:** 1 Division of Biomedical Sciences, University of California Riverside, Riverside, California, United States of America; 2 Institute for Integrative Genome Biology, Center for Disease Vector Research and Department of Entomology, University of California Riverside, Riverside, California, United States of America; 3 Institute for Integrative Genome Biology, Center for Plant Cell Biology, Department of Botany and Plant Sciences, University of California Riverside, Riverside, California, United States of America; University of Illinois at Chicago College of Medicine, United States of America

## Abstract

Mammals and plants share pathogen-sensing systems named nod-like receptors (NLRs). Some NLRs form the inflammasome, a protein scaffold that regulates the secretion of interleukin (IL)-1β and IL-18 by cleaving catalytically inactive substrates into mature cytokines. Here, we show an immune conservation between plant and mammalian NLRs and demonstrate that the murine nuclear receptor binding SET domain protein 1 (NSD1), a protein that bears similarity to the NLR regulator enhanced downy mildew 2 (EDM2) in *Arabidopsis*, diminishes caspase-1 activity during extracellular stimulation with *Listeria monocytogenes* listeriolysin O (LLO). EDM2 is known to regulate plant developmental processes, whereas NSD1 is associated with developmental disorders. We observed that NSD1 neither affects nuclear factor (NF)-κB signaling nor regulates NLRP3 inflammasome gene expression at the chromatin, transcriptional or translational level during LLO stimulation of macrophages. Silencing of *Nsd1* followed by LLO stimulation led to increased caspase-1 activation, enhanced post-translational maturation of IL-1β and IL-18 and elevated pyroptosis, a form of cell death associated with inflammation. Furthermore, treatment of macrophages with LLO^W492A^, which lacks hemolytic activity due to a tryptophan to alanine substitution in the undecapeptide motif, indicates the importance of functional LLO for NSD1 regulation of the NLRP3 inflammasome. Taken together, our results indicate that NLR signaling in plants may be used for gene discovery in mammals.

## Introduction

The inflammasome is a critical component of the innate immune system that provides immediate protection against an infectious insult or cellular damage [1]. The canonical protein scaffold is formed by nod-like (NLRs) or an absent-in-melanoma 2 (AIM2) receptors, the apoptosis-associated speck-like protein (ASC) and caspase-1. Inflammasome activation leads to the release of interleukin (IL)-1β and IL-18 and occurs as a two-tier system [2]. The first signal (priming) involves the activation of the nuclear factor (NF)-κB pathway, which induces the transcription and translation of pro-inflammatory cytokines and other genes. Following this, inflammasome activation results in the maturation of IL-1β and IL-18 by the enzyme caspase-1 [3], [4]. Compared to the classical NLRC4, NLRP1 and AIM2 inflammasomes [5], NLRP3 is uniquely activated by innumerable stimulants, ranging from danger signals to bacterial structures and pore-forming toxins [6].

Owing to the importance of inflammasomes in immune recognition and the response to pathogen infection, numerous groups have examined their activity during exposure to the model pathogen *Listeria monocytogenes* [7]–[10]. Initially studied for its ability to escape vacuoles as a means to promote its dissemination, it has been shown that AIM2, NLRC4, NLRP7 and NLRP3 inflammasomes can recognize *L. monocytogenes* [11], [12]. The intracellular role of the virulence factor listeriolysin O (LLO), encoded by the gene *hly,* has been well characterized. However, its extracellular activities remain mostly unclear [13]. This is surprising because LLO has been used for serodiagnosis of listeriosis patients for decades [14]. Furthermore, a fraction of LLO is functional in the extracellular space [15], and extracellular LLO is important to initiate bacterium internalization, autophagy, and manipulation of histones and post-translational modifications [13].

We drew upon the similarities between plant and mammalian pathogen-sensing systems to address the void in our knowledge regarding the connection between extracellular LLO and inflammasome activity. Previously, we demonstrated that the *Arabidopsis* protein enhanced downy mildew 2 (EDM2) regulated a NLR gene named *Recognition of Peronospora Parasitica 7* (*RPP7*) during oomycete infection in plants [16]. Additionally, EDM2 acted as a substrate for the protein kinase WNK8 [17]. Downstream of WNK8, EDM2 affects the floral repressor FLC to modulate floral transition [17]. More recently, we observed that the mutation of EDM2 altered the activity of the transposons *Mu1* and *COPIA4* [18]. Disruption of dimethylation in histone (H) 3 lysine (K) 9 (H3K9me2) and H3K27me1 levels at the transposon loci influenced development. EDM2 shares similarity with the nuclear receptor-binding SET domain protein 1 (NSD1) in mammals [16]. NSD1 is a lysine methyltransferase that carries co-regulatory domains depending on the presence or absence of a ligand [19], [20]. *Nsd1* plays a role in several pathologies, including but not limited to Sotos and Weaver syndromes, acute myeloid leukemia, breast cancer, neuroblastoma and glioblastoma formation [21]–[25]. NSD1 can also alter transcription by interacting with the protein NSD1-interacting zinc finger protein 1 (NIZP1) [26] and may act as a methyltransferase that preferentially methylates H3 and H4 on K36 and K20 [27], [28].

NSD1 function in infectious diseases has not been thoroughly studied. In this study, we examined the relationship between NSD1 and the NLRP3 inflammasome during extracellular exposure to the cholesterol-dependent cytolysin LLO. Here, we show that the NSD1 regulation of caspase-1 activation during LLO stimulation of macrophages does not influence NF-κB signaling, chromatin dynamics or transcription and translation of inflammasome genes. NSD1 affects the maturation of caspase-1, which in turn modulates IL-1β, IL-18 secretion and a specialized form of cell death referred to as pyroptosis.

## Materials and Methods

### Bioinformatics

Amino acid sequences of PHD fingers were analyzed using ClustalW (http://www.ebi.ac.uk/Tools/msa/clustalw2/) [29]. Protein schematic was obtained from SMART: Simple Modular Architecture Research Tool (http://smart.embl-heidelberg.de/) [30]. Nucleosome prediction for primer design was done using the NuPoP: Nucleosome Positioning Prediction Engine (http://nucleosome.stats.northwestern.edu/) [31]. The caspase-1 gene map was created using Ensembl (http://uswest.ensembl.org/index.html) [32].

### Ethics Statements

All animal breeding and experiments were performed in strict compliance with guidelines set forth by the National Institutes of Health (Office of Laboratory Animal Welfare (OLAW) - Assurance number A3439-01). All animal and biosafety procedures were approved by the Institutional Animal Care and Use (IACUC number: A-20110030BE) and Biological Use Authorization (BUA number: 20120020) Committees at the University of California, Riverside. C57BL/6 mice were purchased from Jackson Laboratories. *Nlrp3* ^−/−^ and *Nlrc4* ^−/−^ mice were obtained from Millennium Pharmaceuticals.

### Cell Culture Generation and Conditions

We used male mice 12–20 weeks of age. Bone marrow-derived macrophages (BMDMs) were generated as previously described with minor modifications [33]. Briefly, femurs and tibias were removed from C57BL/6, *Nlrp*3^−/−^ and *Nlrc*4^−/−^ mice and kept in phosphate buffered saline (PBS)+1% Penicillin-Streptomycin-Amphotericin (PSA) (ThermoScientific). Muscle was removed from femurs and tibias using scissors and razor blades. The ends were cut and marrow was flushed from the bone using cold Dulbecco's Modified Eagle Medium (DMEM) (Invitrogen) with a 27 gauge needle. BMDMs were grown on 10 cm petri dishes in 10 ml of DMEM media supplemented with 10% fetal calf serum (FCS) (Invitrogen), 30% L929 cell conditioning medium, and 1% PSA. BMDMs were grown in a humidified incubator at 37°C with 5% CO_2_ for 6 days prior to stimulation. On the 3^rd^ day, 10 ml of DMEM+10% FCS+30% L929 cell conditioning medium+1% PSA was added to each dish. BMDMs were plated on 24-well culture plates at 1×10^6^ cells per well, unless otherwise stated, in 500 µl of DMEM+10% FCS+1% PSA.

### Macrophage Silencing, Stimulation and Infection

*Nsd1* was silenced with 100 nM of Ambion Silencer Negative Control siRNA #1 (Ambion AM 4635) or Ambion Silencer Pre-designed siRNA for *Nsd1* (Ambion AM 16706) using a 1∶1 ratio of siRNA to Lipofectamine 2000 (Invitrogen). 48 hours after siRNA transfections, BMDMs were primed with 0.1 µg/ml of LPS (Invivogen) for 2 hours. BMDMs were stimulated with 500 ng/ml or 8 µg/ml of LLO for 30 minutes or 1 hour, respectively, as was determined by previous literature, serial dilutions, and hemolytic assays [34], [35]. Treatment with 500 µg/ml of Imject alum (Thermo Scientific) or 10 µM of nigericin (Sigma) was done for 6 hours. Stationary phase *Pseudomonas aeruginosa* PAO1 was used to infect BMDMs at a multiplicity of infection (MOI) of 50 for 1 hour. Wild-type (WT) (10403S), Δ*hly* (DP-L2161), and *L. pneumophila* flagellin (*L.p*. FlaA) (DP-L5964) expressed by *L. monocytogenes* were grown in BD Bioscience Bacto Brain Heart Infusion media overnight at 30°C while kept stationary. Absorbance at OD 595 nm was measured and values between 1.2–1.4 were used. Cultures were diluted (1∶10) with sterile PBS (Thermo). BMDMs were infected using MOI 10. 30 minutes after infection, media was replaced with 50 µg/ml Gentamicin/Amphotericin B (Cascade Biologics)+DMEM.

### Recombinant LLO and LLO^W492A^ Expression

*Escherichia coli* strain BL21 (DE3) carrying plasmid pET29:6xHis-LLO or pET29b-LLO W492A-His6 was used to express recombinant LLO and LLO^W492A^, respectively. Expression and purification was done as described with minor modifications [36]. *E. coli* was grown with agitation, at 37°C overnight, in 10 ml of LB broth (Teknova) supplemented with 50 µg/ml of kanamycin (Sigma). The following day, 100 ml of Luria Bertani (LB) broth was added and 50 µg/ml of kanamycin was supplemented. Expression was induced by isopropyl-β-D-thiogalactopyranoside (IPTG) from Sigma. Cultures continued to grow at 30°C for 18 hours with agitation. *E. coli* was pelleted (4,000×g, 15 minutes, 4°C). The pellet was resuspended in 1 ml of lysis buffer (50 mM Na2HPO4, 300 mM NaCl, 1 mM phenylmethylsulfonyl fluoride (PMSF), 10 mM imidazole). The pelleted expression culture was sonicated 4 times (20% power, 15 second pulses, 1 minute rests on ice) (VWR Scientific Branson Sonifier 450). Purification was done using a Qiagen Ni-NTA spin column. Purification was done as recommended by Qiagen. Lysates were centrifuged in columns for 5 minutes at 270×g. Spin columns were washed with wash buffer (50 mM Na2HPO4, 300 mM NaCl, 1 mM PMSF, 20 mM imidazole). A 16% glycerol wash (16% glycerol and wash buffer) and a high NaCl wash (700 mM NaCl and wash buffer) were performed. A rinse with wash buffer was done after each wash. Proteins were eluted twice into an elution buffer (0.136 g of imidazole and wash buffer). All washes and elution were centrifuged at 700×g for 2 minutes at 4°C. Eluate was concentrated with Millipore Amicon Ultra 3000 MWCO filter unit. Recombinant LLO was kept at −80°C in a storage buffer (10 mM 4-(2-hydroxyethyl)-1-piperazineethanesulfonic acid (HEPES), 140 mM NaCl, 1 mM ethylenediaminetetraacetic acid (EDTA)). Several batches of recombinant LLO were expressed and purified. Variability between lots resulted in the adjustment of concentrations used.

### Immunofluorescence Microscopy

BMDMs were cultured as described above. 2×10^6^ cells were grown on 18 mm glass coverslips in 6 well plates. Cells were stimulated with tumor necrosis factor (TNF)-α (50 ng/ml) and LLO (500 ng/ml). Cells were washed twice with PBS and fixed with methanol. A 1∶200 dilution of a custom made NSD1 antibody (Fisher Scientific) was used. Coverslips were incubated in the primary antibody for 1 hour at room temperature. Slips were washed and incubated in an anti-rabbit fluorescence-conjugated secondary antibody (Millipore) at room temperature for 30 minutes. Slips were mounted onto a slide using Vectashield mounting media with 4′,6-diamidino-2-phenylindole (DAPI). Confocal microscopy was done with a Leica SP2. Original magnification was 63× with an enlargement of 4x.

### Quantitative Real-Time RT-PCR (qPCR)

RNA extraction was done with TRIzol (Invitrogen). First strand cDNA was synthesized using Verso cDNA kit purchased from Thermo Scientific. qPCR was done with iQ SYBR Green Supermix, on either a Bio-Rad iQ5 or MyiQ real-time PCR detection system, and data was processed by iQ5 software from Bio-Rad. Data was analyzed by the ΔΔC_T_ method [37]. β*-*actin was used as the normalizing control. Primer sequences were as follows: for β*-*actin-F (5′-CGCATCCTCTTCCTCCCT-3′) and β*-*actin-R (5′-TGGAATCCTGTGGCATCC-3′); caspase-1 a-F (5′-CAACCATTCCTTGGTCCACT-3′) and caspase-1 a-R (5′-ATTGATGTGGGGGAAAGGTT-3′); caspase-1 b-F (5′-TACCTGGCAGGAATTCTGGA-3′) and caspase-1 b-R (5′-GCAGAGCCACAGACACAAAA-3′); caspase-1 c-F (5′-CCTACCAGCATTTCAGGCATA-3) and caspase-1 c-R (5′-TGTTGGCTGTAGGTGTGGAA-3′); *Nlrp3* (a)- F (5′-TTATGTTGGACTGGGCACTG-3′) and *Nlrp3* (a)-R (5′-ATCAAAGCCATCCATGAGGA-3′); *Nlrp3* (b)-F (5′-CCCCATTACCTAACCCCATC-3′) and *Nlrp3* (b)-R (5′-GGAAATTCTGATGTACCTG AACAC-3′); *Asc*-F (5′-TGTCAGGGGATGAACTCAAA-3′) and *Asc*-R (5′-CAGCTCCTG TAAGCCCATGT-3′); *Nsd1*-F (5′-ACCTGACAGAGCCTCTCCAA-3′) and *Nsd1*-R (5′-GCTGGAGTTTTCTCCACTGC-3′); *caspase-11*- F (5′- ACGATGTGGTGGTGAAAGAGGAGC- 3′) and *caspase-11-* R (5′- TGTCTCGGTAGGACAAGTGATGTGG-3′). β-actin, *Asc*, *Nlrp3* (a), *Nsd1*, and caspase-1 c primers were used for qPCR. All caspase-1 primers and *Nlrp3* (b) were used for chromatin immunoprecipitation (ChIP)-qPCR. Primers were designed using Life Technologies OligoPerfect Designer (http://tools.invitrogen.com).

### Enzyme-Linked Immunosorbent Assay (ELISA)

ELISAs were performed for the detection of IL-1β, IL-18, and IL-6 using BD OptEIA kits from BD Biosciences. Supernatants used were collected from cells cultured and stimulated in all experiments. Absorbance was measured using Bio-Rad iMark at 450 nm with a 595 nm correction.

### Immunoblotting

Total cell lysates from 24 well plates cultured and stimulated, as described previously, were extracted using radioimmunoprecipitation (RIPA) lysis buffer (Boston Bioproducts) with Complete Mini Protease Inhibitor Cocktail and PhosSTOP, both from Roche Applied Science. Protein concentration was determined via the Bradford protein assay method, using protein assay dye reagent concentrate and iMark reader, both from Bio-Rad. Either an 8% or 15% SDS polyacrylamide gel was made and ran at 200 volts for 1 hour. Transfer was done in wet conditions with polyvinylidene fluoride (PVDF) membranes for 60–90 minutes at 100 volts. Membranes were blocked in 5% non-fat dry milk (LabScientific, Inc.). Western blot antibodies for NSD1 (Santa Cruz and Custom made Fisher Scientific) (1∶500 and 1∶625), β-actin (Neomarker-Thermo Scientific) (1∶500 and 1∶1000), caspase-1 (Millipore) (1∶500), lamin B1 (Abcam) (1∶100), p-IκB-α (Cell Signaling) (1∶250), NLRP3 (Abcam) (1∶500), ASC (Enzo) (1∶250), pro-IL-1β (R&D) (1∶1000) and caspase-11 (Sigma) (1∶500). Enhanced chemiluminescence (ECL) western blotting substrate and super signal West Pico Chemiluminescent substrate were used to image the blots (Pierce Thermo Scientific).

### Nuclear Protein Extraction

5×10^6^ BMDMs were silenced and stimulated as indicated above. Nuclear protein was extracted using the G-Biosciences: Nuclear and Cytoplasmic Extraction Kit (Catalog# 786–182). The protocol was scaled down appropriately and extraction was done according to the protocol provided. Complete Mini Protease Inhibitor Cocktail (Roche Applied Science) was used.

### Transcription and Degradation Inhibition

Transcription and degradation inhibition was done as previously described [38]. Inhibition of transcription was accomplished using 5 µg/ml of actinomycin D (Sigma-Aldrich). Proteasomal degradation inhibition was done using 1 µM of the reversible proteasome inhibitor MG-132 (Calbiochem).

### Fluorescent Labeled Inhibitor of Caspases (FLICA)

Cells were cultured and stimulated as stated above. Green FLICA Caspase-1 Assay Kit (Catalog #98) was from Immunochemistry and the provided protocol was used. Cell counting was done with BD Biosciences FACSCanto Flow Cytometer. Data was processed using BD FACSDiva Software (BD Biosciences).

### ChIP Assay

BMDMs (45×10^6^) were seeded onto a 15 cm dish. Cells were silenced and primed as previously described. Cells were stimulated with 8 µg/ml of recombinant LLO. After 1 hour of stimulation, the Active Motif ChIP-IT Express kit (Catalog# 53008) was used to prepare chromatin. Briefly, cells were fixed with a fixation solution (1% Sigma formaldehyde final concentration and DMEM). Fixation was stopped with a glycine stop-fix solution. Cells were dounced on ice with 20 strokes of rod A and 20 strokes of rod B prior to sonication. The cell lysate was sonicated (Fisher Scientific Sonic Dismembrator Model 100) with 5 pulses at power 6 for 15 seconds in 700 µl of shearing buffer. DNA was cleaned up, as suggested, in order to assess shearing efficiency. 3 µg of NSD1 (Custom made Fisher Scientific), histone 3 (H3), histone 3 lysine 36 dimethylation (H3K36me2), histone 3 lysine 36 trimethylation (H3K36me3) and the negative control goat anti-rabbit HRP antibodies (all from Abcam) were used per ChIP. Reactions were incubated at 4°C for 4 hours. Magnetic bead-antibody complexes were washed twice in 800 µl of ChIP Buffer 1 and three times with 800 µl of ChIP Buffer 2. After chromatin elution, reverse cross-linking and protein degradation was done. The Qiaquick PCR Purification kit from Qiagen (Catalog# 28104) was used to purify samples. qPCR was used to analyze enrichment as stated above. Fold change and standard errors were determined using the following ΔΔC_T_ protocol: http://www.protocol-online.org/biology-forums/posts/29733.html.

### Hemolytic Assay

10% sheep red blood cells (RBC) were obtained from Lampire Biological Laboratory. A suspension of 0.2% RBC was made by washing RBC three times by centrifuging the suspension at 1500 rpm for 15 minutes at 4°C. 1% Triton X was used as a positive control and PBS was used as a negative control. A serial dilution of the toxin was created, added to the RBC and incubated for 5 minutes on ice. Samples were then incubated for 30 minutes at 37°C and the absorbance was measured at 450 nm.

### LDH Assay

1×10^6^ BMDMs (WT or *Nlrp3* ^−/−^) were transfected with control silencer or *Nsd1* siRNA. BMDMs were primed and untreated or treated with 8 µg/ml WT LLO or LLO^W492A^ for 1 hour. Medium alone was used as the negative control and 1% Triton-X was used as the positive control. Supernatant from controls and samples were reserved for the assay. Percent of LDH release was assayed using the Takara LDH cytotoxicity detection kit. Assay was conducted as recommended. Absorbance was measured using Bio-Rad iMark at 450 nm.

### Statistical Analysis

All graphs were generated in GraphPad Prism 5 and *P* values were calculated using Student's t test. A *P* value of less than 0.05 was considered statistically significant.

## Results

### LLO stimulation of macrophages upregulates NSD1

**Figure 1:**
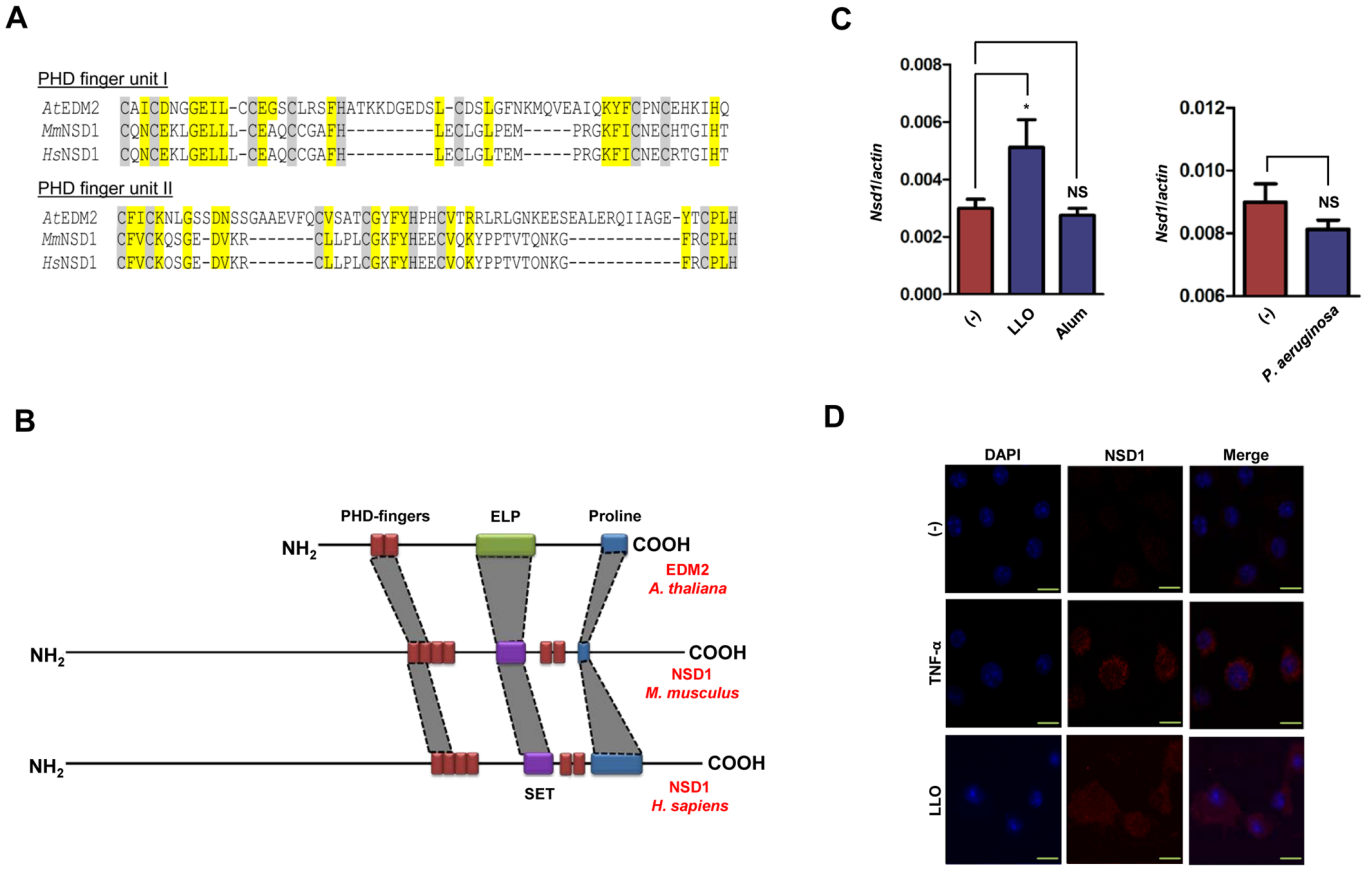
Nsd1 is upregulated during LLO stimulation of macrophages. (A) Displayed peptide stretches are directly adjacent to each other and cover two repeated PHD finger units. Consensus sequences of both PHD finger units based on similarities between *Arabidopsis* (*At*) EDM2 and NSD1 in mice (*Mm*) and humans (*Hs*). Cys or His residues of the conserved zinc coordinating C4HC3 pattern of PHD fingers are highlighted in grey. The last C of the C-terminal PHD finger unit is replaced by H in EDM2/NSD1-type proteins. Other residues conserved between EDM2 and NSD1 are highlighted in yellow. (B) PHD fingers from EDM2 and NSD1 are shown in red. Methyltransferase domains in EDM2 (ELP) and NSD1 (SET) are shown in green and purple, respectively. A proline rich region in both NSD1 and EDM2 is shown in blue. (C) BMDMs (1×10^6^) from C57BL/6 mice were stimulated with (C, *left*) LLO (500 ng/ml) (n = 4) for 30 minutes and alum (500 µg/ml) (n = 4) for 6 hours and (C, *right*) *P. aeruginosa* (MOI 50) (n = 6) for 1 hour. *Nsd1* transcription was evaluated by qPCR and analyzed by the ΔΔC_T_ method. β-actin was used as a normalizing control. Student's t test; (*) *P*<0.05 compared to (−) non-stimulated cells. NS – not significant. (D) Confocal microscopy of BMDMs from C57BL/6 mice untreated or treated with TNF-α (100 ng/ml) or LLO (500 ng/ml). BMDMs were stained with DAPI (blue) and anti-NSD1 (red). Original magnification 63×with an enlargement of 4x. Scale bar = 10 µm. Experiments were repeated at least three times.

Since *L. monocytogenes* LLO is important for bacterial virulence [39], [40] and LLO extracellular activities remain mostly unclear [13], [41], we decided to investigate how host signaling recognizes this bacterial toxin. Owing to the conservation of NLR pathways in plants and mammals, we drew upon the similarities between these two eukaryotic kingdoms for our study. Previously, we identified EDM2 in *Arabidopsis* as a regulator of RPP7, a NLR that mediates recognition of a plant pathogen [16]. EDM2 bears similarity to the mammalian protein NSD1 (Fig. 1A and B). An *in silico* analysis of EDM2 and NSD1 revealed that they possess PHD finger domains that have many conserved residues (Fig. 1A). PHD fingers are involved in nuclear protein-protein interaction. Typically, zinc coordinating sites within PHD fingers carry a featured C4HC3 pattern [42], [43]. The EDM2 and NSD1 C-terminal PHD finger units are characterized by a conserved C4HC2H structure (Fig. 1A). In addition to the PHD fingers, EDM2 and NSD1 share other domains, such as a C-terminal proline-rich region which is thought to play a role in protein-protein interactions and/or transcriptional activation (Fig. 1B) [17]. Furthermore, while NSD1 bears a Su(Var)3-9, Enhancer-of-zeste, Trithorax (SET) methyltransferase domain, EDM2 features an EDM2-like protein (ELP) domain which is likely to have methyltransferase activity [16], [44].

We then proceeded to establish a relationship between NSD1 and caspase-1 activation, since it is known that EDM2 plays a role in mediating the activities of an *Arabidopsis* NLR-containing protein named RPP7 [16] and LLO is recognized by the NLRP3 inflammasome [10]. We stimulated BMDMs with LLO, alum, and *Pseudomonas aeruginosa*, which are agonists for the NLRP3 or the NLRC4 inflammasomes, and evaluated transcription levels of *Nsd1* by qPCR. We noticed an increase in *Nsd1* transcript levels when cells were exposed to LLO, an NLRP3 stimulant that induces inflammasome activation via K^+^ efflux [45] (Fig. 1C, *left*). This effect was not apparent with alum, a particulate agonist of the NLRP3 inflammasome that stimulates caspase-1 via lysosomal disruption and cathepsin B release [46] (Fig. 1C, *left*). Similarly, we did not detect an effect on *Nsd1* transcription during macrophage stimulation with *P. aeruginosa,* a NLRC4 agonist [46] (Fig. 1C, *right*). We then used confocal microscopy to elucidate the impact of LLO on NSD1 protein levels. Similar to our results observed with *Nsd1* transcription, the levels of NSD1 also increased with TNF-α, a positive control, and LLO treatment compared to untreated cells (Fig. 1D). With these experiments, we concluded that *Nsd1* expression increases when mouse macrophages are exposed to the NLRP3 agonist LLO.

### LLO activates the NLRP3 inflammasome and promotes IL-1β secretion

**Figure 2:**
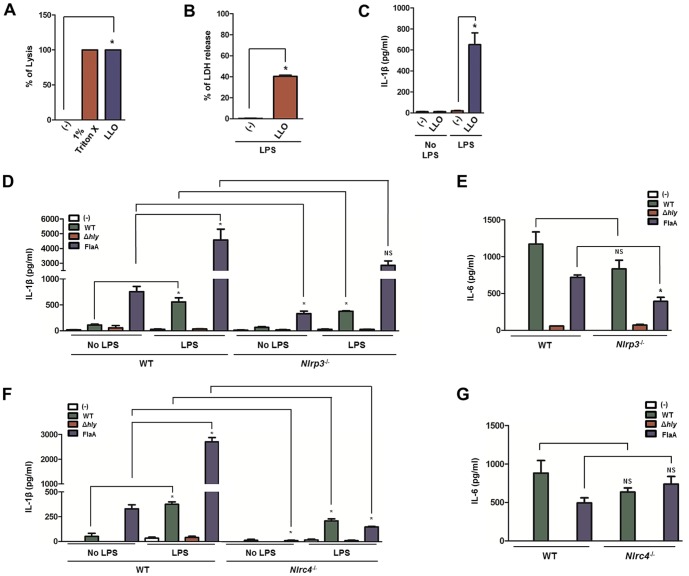
Listeriolysin O is recognized by the NLRP3 inflammasome. 1×10^6^ BMDMs were primed with LPS (100 ng/ml) for 2 hours. BMDMs were treated with recombinant LLO (8 µg/ml for hemolysis and LDH or 500 ng/ml for IL-1β) or infected with the *L. monocytogenes* WT, Δ*hly*, or *L.p.*FlaA strains MOI 10 for 6 hours after gentamicin (50 µg/ml) medium replacement at 30 minutes. (A) Hemolysis, (B) LDH, and (C) IL-1β were measured for recombinant LLO. (D and F) IL-1β secretion by WT (n = 4), *Nlrp3^−^* ^/−^ (n = 4) and *Nlrc4* ^−/−^ macrophages (n = 4) was analyzed by ELISA. (E and G) Stimulation was repeated, as previously stated, without priming and IL-6 secretion by WT (n = 4), *Nlrp3^−^* ^/−^ (n = 4) and *Nlrc4* ^−/−^ macrophages (n = 4) was determined. Student's t test; (*) *P*<0.05, WT compared to either unprimed or knockout. NS – not significant. Experiments were repeated at least twice.

LLO is known for its key cytolysin feature [47]. It possesses efficient hemolytic abilities (Fig. 2A). Cellular lysis is frequently linked with cell death and here we observed that LLO induces macrophage cell death [48] (Fig. 2B). Moreover, recombinant LLO is a clear inducer of IL-1β secretion (Fig. 2C). To examine the effect of *L. monocytogenes*-derived LLO on IL-1β secretion and inflammasome activity in macrophages, we infected bone marrow-derived macrophages (BMDMs) with WT and a *L. monocytogenes* strain lacking the virulence factor LLO (here described as Δ*hly L. monocytogenes*) [11]. As a positive control, we used a genetically engineered strain of *L. monocytogenes* that expresses *Legionella pneumophila* flagellin (*L.p*. FlaA), a strong inducer of the NLRC4 inflammasome [49]. In the absence of priming, minimal levels of IL-1β were secreted by WT, *Nlrp3^−/−^* and *Nlrc4^−/−^* macrophages during infection with WT and Δ*hly L. monocytogenes* (Fig. 2D and F). However, unprimed WT macrophages stimulated with the *L.p*. FlaA strain were able to secrete moderate levels of IL-1β, most likely due to the effects of flagellin on Toll-like receptor (TLR) 5 and NLRC4 recognition. Although TLR5 and NLRC4 both recognize flagellin, TLR5 distinctly identifies the D1 region of flagellin on the cell surface [50], while NLRC4 detects the C-terminus of the D0 region of this bacterial component intracellularly [46], [51]. Hence, *L. pneumophila* flagellin may act to prime the production of immature IL-1β via TLR5 and induce maturation via caspase-1 activation.

After priming, WT macrophages secreted IL-1β during WT *L. monocytogenes* infection and, more so, with the *L.p*. FlaA strain (Fig. 2D and F). Confirming functionality, *Nlrc4* ^−/−^ macrophages demonstrated a deficiency in IL-1β secretion when infected with the *L.p*. FlaA strain (Fig. 2F). Even though less pronounced, *Nlrc4^−^* ^/−^ macrophages also secreted lower levels of IL-1β during infection with WT *L. monocytogenes*, most likely due to the lack of endogenous *L. monocytogenes* flagellin recognition by NLRC4. In all instances, treatment with *L. monocytogenes* lacking *hly*, the gene that codes for LLO, resulted in negligible amounts of IL-1β secretion (Fig. 2D and F). When contrasting primed WT and *Nlrp3* ^−/−^ macrophages, we observed a statistically significant decrease in IL-1β secretion in cells exposed to WT but not the *L.p*. FlaA strain (Fig. 2D). The decrease in IL-1β secretion from *Nlrp3* ^−*/−*^ cells treated with the WT *L. monocytogenes* strain could potentially be due to the absence of LLO detection by NLRP3.

IL-6 was measured to determine the effect of *Nlrp3* or *Nlrc4* deficiency on caspase-1 independent signaling pathways. WT and *L.p*. FlaA *L. monocytogenes* elicited IL-6 from WT macrophages, whereas *L. monocytogenes* Δ*hly* was unable to induce IL-6 secretion (Fig. 2E and G). The absence of *Nlrc4* or *Nlrp3* did not affect IL-6 secretion by macrophages during stimulation with WT *L. monocytogenes*. On the other hand, *Nlrp3* ^−*/−*^ macrophages slightly altered IL-6 levels after infection with the *L.p*. FlaA strain (Fig. 2E). It is unclear why IL-6 levels were moderately affected by the *L.p*. FlaA strain in *Nlrp3* ^−/−^ macrophages. However, it has been shown that IL-6 signaling may be affected by IL-1β secretion downstream of caspase-1-dependent signaling pathways [52], [53]. Our results suggest that: (1) WT *L. monocytogenes* elicits low levels of IL-1β secretion; and (2) flagellin and LLO are seemingly important for NLRC4 and NLRP3 recognition in mouse macrophages.

### NSD1 restricts NLRP3 inflammasome-mediated cytokine secretion during LLO stimulation of macrophages

**Figure 3:**
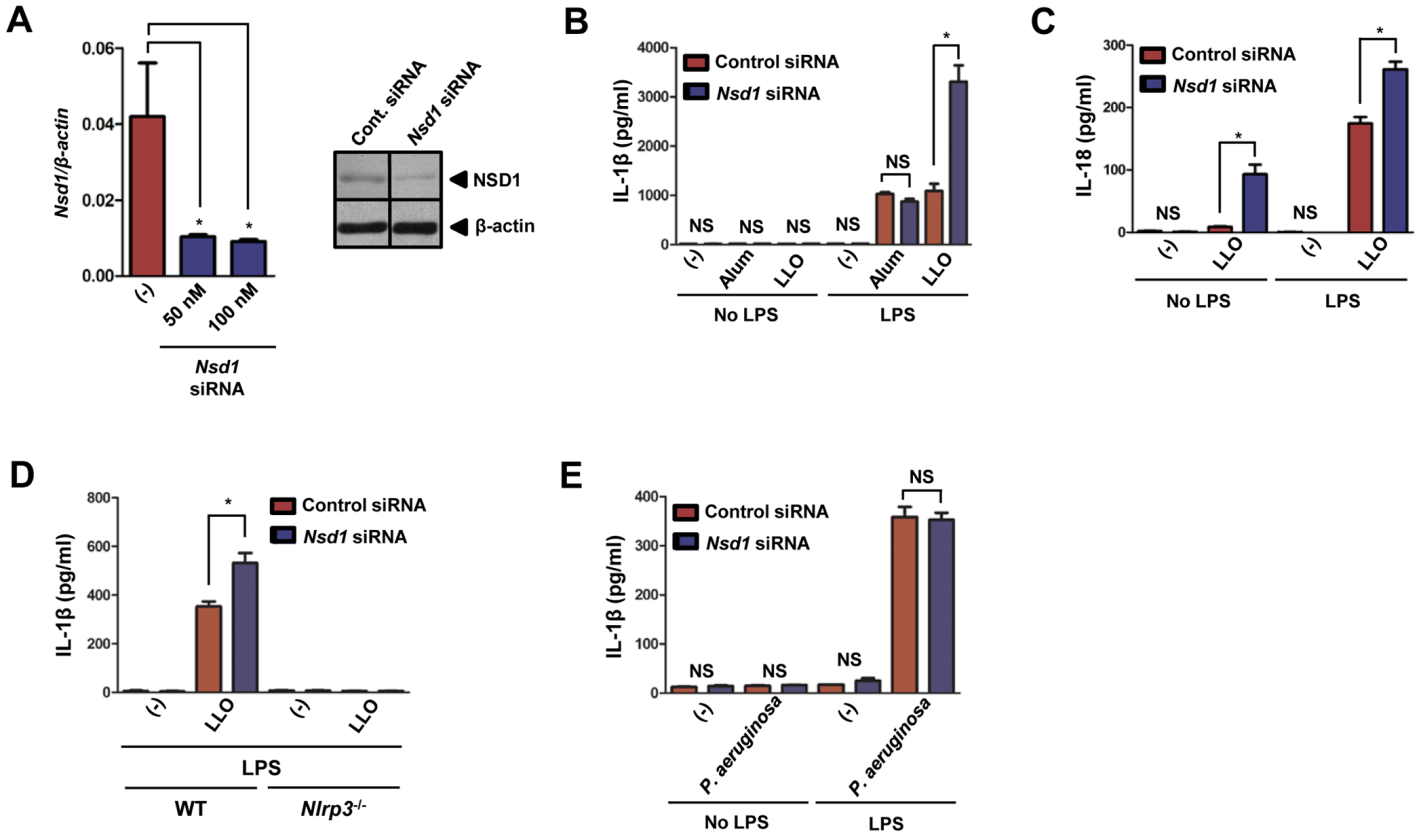
NSD1 inhibits LLO-mediated secretion of IL-1β and IL-18 by macrophages . (A, *left*) 50 and 100 nM of *Nsd1* siRNA successfully reduced *Nsd1* transcription at 48 hours (n = 6). Student's t test; (*) *P*<0.05 compared to non-transfected cells. (A, *right*) 100 nM of *Nsd1* or control silencer siRNA was transfected into BMDMs (1×10^6^) by using Lipofectamine 2000. Cell lysate was immunoblotted for NSD1. β-actin was used to verify equal loading. 1×10^6^ BMDMs were primed with LPS (100 ng/ml) and treated with control silencer or *Nsd1* siRNA. Also, BMDMs were untreated or stimulated with LLO (500 ng/ml) for 30 minutes. (B) IL-1β (n = 6) and (C) IL-18 (n = 6) were measured by ELISA after NLRP3 (500 ng/ml LLO or 500 µg/ml alum) stimulation. (D) After priming with LPS and stimulation with 8 µg/ml recombinant LLO, IL-1β levels secreted by BMDMs from WT (n = 3) and *Nlrp3* ^−/−^ (n = 4) mice were measured by ELISA. (E) IL-1β was measured by ELISA after NLRC4 stimulation (*P. aeruginosa* MOI 50) (n = 4). Student's t test; (*) *P*<.05 compared to cells transfected with control siRNA. NS – not significant. Experiments were repeated at least twice.

Due to the embryonic lethality of *Nsd1* knockout mice [28], we used siRNA-mediated silencing to study the effects of NSD1 on innate immunity. We tested both 50 and 100 nM of siRNA in our experimental design and discovered that these concentrations resulted in substantial reduction of *Nsd1* expression in BMDMs at 48 hours post-transfection (Fig. 3A, *left*). This decrease was confirmed by immunoblotting, which revealed a 50% decrease of NSD1 in the cells given *Nsd1* siRNA (Fig. 3A, *right*). IL-1β and IL-18 were analyzed to determine if NSD1 influences the maturation of these pro-inflammatory cytokines (Fig. 3B-C). Secretion of IL-1β and IL-18 increased with *Nsd1* reduction and LLO stimulation of macrophages. This effect seemed specific for LLO because alum, another NLRP3 inflammasome stimulant, did not affect IL-1β release when *Nsd1* was silenced in macrophages (Fig. 3B). Furthermore, we observed that NLRP3 is crucial during LLO stimulation because *Nlrp3* ^−/−^ macrophages exhibited abolishment of IL-1β secretion (Fig. 3D). As a negative control, NLRC4-mediated IL-1β secretion was measured and observed to be unaffected when *P. aeruginosa* was used to activate the inflammasome in the presence of normal and reduced levels of NSD1 (Fig. 3E). Our findings suggest the specificity of NSD1 regulation on the NLRP3 inflammasome in the presence of LLO.

### NSD1 does not affect NF-κB signaling in response to LLO stimulation of macrophages

**Figure 4:**
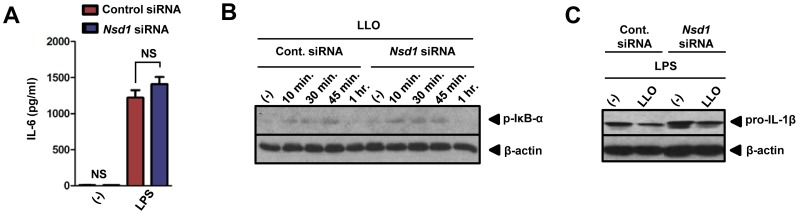
NSD1 does not affect the NF-κB signaling pathway in macrophages . 1×10^6^ BMDMs were transfected with control silencer or *Nsd1* siRNA. After 48 hours, BMDMs were untreated (−) or treated overnight with LPS (500 ng/ml). (A) IL-6 was measured by ELISA (n = 8). Student's t-test; (*) *P*<0.05 compared to cells given control siRNA. NS – not significant. (B) BMDMs (1×10^6^) were untreated or stimulated with LLO (500 ng/ml). Cell lysates were collected at the indicated time points. Immunoblotting was performed to determine levels of p-IκB-α. β-actin was used to determine equivalent loading. (C) 1×10^6^ BMDMs were primed with LPS (100 ng/ml) and treated with control silencer or *Nsd1* siRNA. Also, BMDMs were untreated or stimulated with LLO (500 ng/ml) for 30 minutes. Cell lysates were immunoblotted for pro-IL-1β. β-actin was used to determine equal loading. Experiments were repeated at least twice.

Since the NF-κB pathway regulates transcription of inflammatory genes [54] and LLO stimulates the NLRP3 inflammasome [10], we measured the amount of IL-6 for LPS and LLO treated cells after siRNA transfection. Diminished levels of NSD1 did not influence the secretion of LPS-induced IL-6 secretion from macrophages (Fig. 4A). A negligible amount of IL-6 was released from LLO-stimulated cells (*data not shown*). Further analysis, using a time course experiment visualizing p-IκB-α, a read-out for NF-κB activation [55], showed similar levels of NF-κB activation in non-silenced and *Nsd1* silenced cells given LLO (Fig. 4B). In both treatments, phosphorylation of IκB-α initiated 10 minutes after stimulation with LLO and returned to pre-treatment levels after 45 minutes. Further confirming that NSD1 regulatory activity is independent of signal 1, pro-IL-1β was measured by western blot. After LPS priming, pro-IL-1β levels remained relatively constant in silenced and non-silenced cells (Fig. 4C). These results suggested that the NSD1 role during LLO stimulation of macrophages was independent of the NF-κB pathway.

### NSD1 does not restrict NLRP3 inflammasome activation at the chromatin level

**Figure 5:**
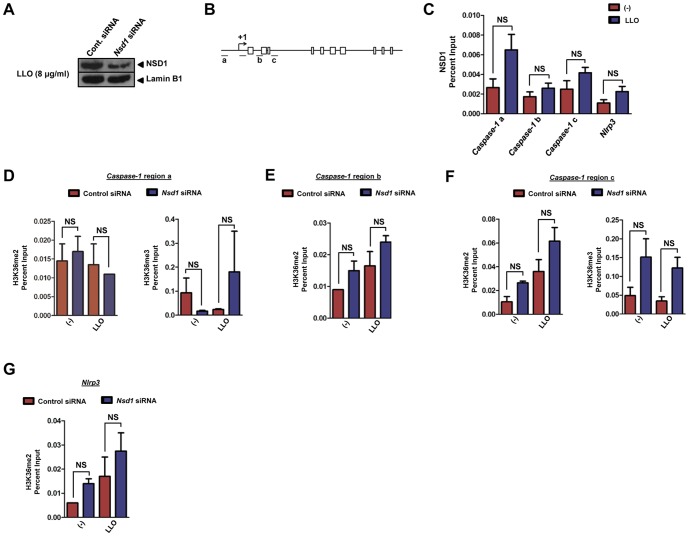
NSD1 does not impart chromatin modifications at the 5′ end of caspase-1. BMDMs (5×10^6^) were transfected with 100 mM of *Nsd1* or control silencer siRNA using Lipofectamine 2000 (n = 6). Following 48 hours, BMDMs were primed with LPS (100 ng/ml) for 2 hours and treated with 8 µg/ml of LLO for 1 hour. Nuclear proteins were isolated from whole cell lysates and an (A) immunoblot was performed for NSD1. Lamin B1 was used to determine equal loading. BMDMs (45 ×10^6^) were treated as previously described in (A). (B) Regions of caspase*-1* which were analyzed are as follows: (a) -1000 bp upstream of the transcription start site (TSS), (b) +1000 bp downstream of the TSS, and (c) +2000 bp downstream of the TSS. Boxes indicate exons. (C) ChIP was performed for NSD1 followed by qPCR for the indicated regions of caspase*-1* and *Nlrp3*. *Nlrp3* was measured as a negative control. ChIP-qPCR data is represented as mean+SE. A graph was chosen to be representative of two experiments. Additionally, BMDMs (45×10^6^) were transfected with 100 mM of *Nsd1* or control silencer siRNA using Lipofectamine 2000. Following 48 hours, BMDMs were primed with LPS (100 ng/ml) for 2 hours and treated with 8 µg/ml of LLO for 1 hour. H3K36me2 was analyzed for each region of caspase*-1* by ChIP-qPCR (n = 2): (D, *left*) region a, (E) region b, and (F, *left*) region c. H3K36me3 was also measured for (D, *right*) region a and (F, *right*) region c. (G) H3K36me2 of *Nlrp3* was determined as a negative control. ChIP-qPCR data is represented as mean+SE. Student's t test; *P*<0.05 compared to cells transfected with control siRNA. NS – not significant.

Earlier work demonstrated the ability of NSD1 to act as a histone methyltransferase [56] and has indicated that NSD1 targets the 5′ end of genes [28]. Therefore, we investigated whether any NSD1-mediated chromatin modifications were associated with NLRP3 inflammasome regulation during LLO stimulation of macrophages. First, we used a prediction engine named NuPoP to determine nucleosome positioning in the NLRP3 inflammasome genes. We then silenced *Nsd1* and observed, by western blot, a 50% reduction of nuclear NSD1 during LLO stimulation of macrophages (Fig. 5A). Utilizing chromatin immunoprecipitation (ChIP), we observed that areas corresponding to the region -1000 bp to +2000 bp from the transcription start site (TSS) of caspase-1 were not differentially occupied by NSD1 during stimulation with LLO (Fig. 5B-C). Initially, our experiments included the analysis of the TSS; however, NSD1 binding at the +1 site was highly variable and could not be represented. Physical association of NSD1 with *Nlrp3* and *Asc* was also quantified and did not show enrichment after treatment with LLO in *Nsd1*-silenced versus non-silenced cells (Fig. 5C, *data not shown*).

To further elaborate our findings, we investigated whether the histone marks associated with three regions previously analyzed (−1000 bp, +1000 bp, and +2000 bp from the caspase-1 TSS) would be altered upon stimulation. NSD1 regulates the methylation status of histone 3 lysine 36 (H3K36) and histone 4 lysine 20 (H4K20). However, increased specificity has been reported for H3K36 [22], [27], [44]. Our experiments showed dimethylation (me2) and trimethylation (me3) of histone 3 lysine 36 (H3K36) at sites along the caspase-1 locus (Fig. 5D-F). Overall, a difference in enrichment between cells transfected with control or *Nsd1* siRNA was not observed. Dimethylation of *Nlrp3* was used as a negative control (Fig. 5G). Trimethylation of the caspase-1 locus region b and *Nlrp3* were not represented because of the lack of consensus between ChIP-qPCR experiments. Taken together, our observations support the findings that NSD1 does not regulate the NLRP3 inflammasome genes at the chromatin level.

### The NLRP3 inflammasome gene expression in macrophages was not influenced by NSD1 during stimulation with LLO

**Figure 6:**
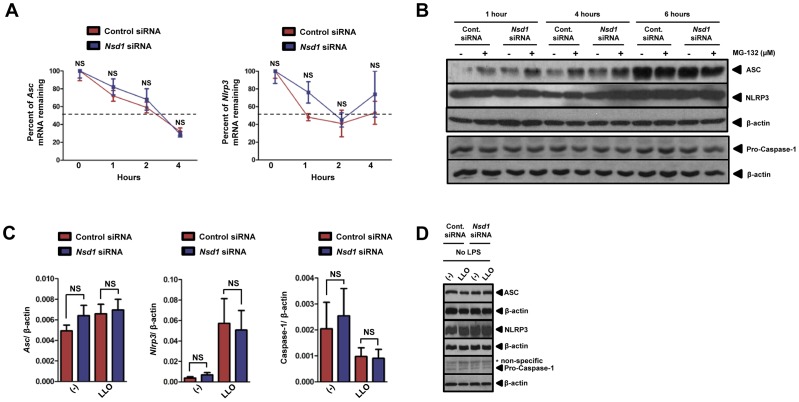
Nsd1 silencing does not alter Asc, Nlrp3, and caspase-1 gene expression. BMDMs (1×10^6^) were transfected with 100 nM of *Nsd1* or control silencer siRNA using Lipofectamine 2000. BMDMs (n = 5) were treated with actinomycin D (5 µg/ml) followed by qPCR analysis of remaining mRNA for (A, *left*) *Asc* and (A, *right*) *Nlrp3*. Dashed line indicates 50% of mRNA remaining. (B) Silenced and non-silenced BMDMs were either untreated (−) or treated (+) with 1 µM of MG-132, a reversible proteasomal inhibitor. ASC, NLRP3, and pro-caspase-1 levels were determined by immunoblotting. β-actin was used to confirm equal loading. (C) 48 hours after silencing, BMDMs were stimulated with LLO (500 ng/ml) for 30 minutes (n = 8). (C, *left*) *Asc*, (C, *center*) *Nlrp3,* and (C, *right)* caspase-1 transcription was evaluated by qPCR. qPCR was analyzed using the ΔΔC_T_ method. Student's t test; *P*<0.05 compared to cells transfected with control siRNA. NS – not significant. (D) Silenced and non-silenced BMDMs were either non-treated or stimulated with LLO (500 ng/ml) for 30 minutes. Immunoblot was performed for ASC, NLRP3, pro-caspase-1. Equal loading was determined using β-actin. * – non-specific bands. Experiments were repeated at least twice.

We then examined the transcriptional and translational levels of NLRP3, ASC and caspase-1. We first used the transcription inhibitor actinomycin D to determine whether NSD1 affected the mRNA stability of *Nlrp3* and *Asc*. *Asc* and *Nlrp3* transcript levels were measured by qPCR after transfection with control and *Nsd1* siRNA. The half-life of mRNA from both genes was found to be similar at approximately 2 hours (Fig. 6A). Overall, the percentage of *Asc* and *Nlrp3* mRNA remaining from silenced and non-silenced cells stayed consistent after the addition of the transcriptional inhibitor. Translation of ASC, NLRP3, and the effector enzyme precursor pro-caspase-1 was analyzed by immunoblot in the presence or absence of 1 µM of the proteasomal degradation inhibitor MG-132 (Fig. 6B). At each time point, there were similar levels of ASC in the control and *Nsd1* siRNA transfected cells when the proteasomal inhibitor MG-132 was used. However, moderate protein levels of ASC were observed when *Nsd1* was silenced and compared to control siRNA at 1 and 4 hours in the absence of MG-132. Next, we observed increased protein levels of NLRP3 for 6 hours. Neither *Nsd1* silencing nor proteasomal degradation affected NLRP3 translation. Pro-caspase-1 was also not affected by any conditions used in our analysis (Fig. 6B).

*Asc*, *Nlrp3*, and caspase-1 transcription and translation were also measured after stimulation with LLO. With the exception of a slight increase of *Nlrp3* transcript levels and reduction in the caspase-1 transcript levels in the presence of LLO, *Nlrp3*, *Asc* and caspase-1 did not exhibit significant transcriptional changes regardless of the addition of *Nsd1* siRNA (Fig. 6C). Our observation with *Nsd1* silencing was supported by the analysis of ASC, NLRP3, and pro-caspase-1 proteins, which remained consistent despite treatment and *Nsd1* reduction (Fig. 6D). Overall, any potential effects of NSD1 on NLRP3 inflammasome genes were most likely not through transcription and translation.

### LLO pore formation is necessary for NSD1 restriction of caspase-1 activation in macrophages

**Figure 7:**
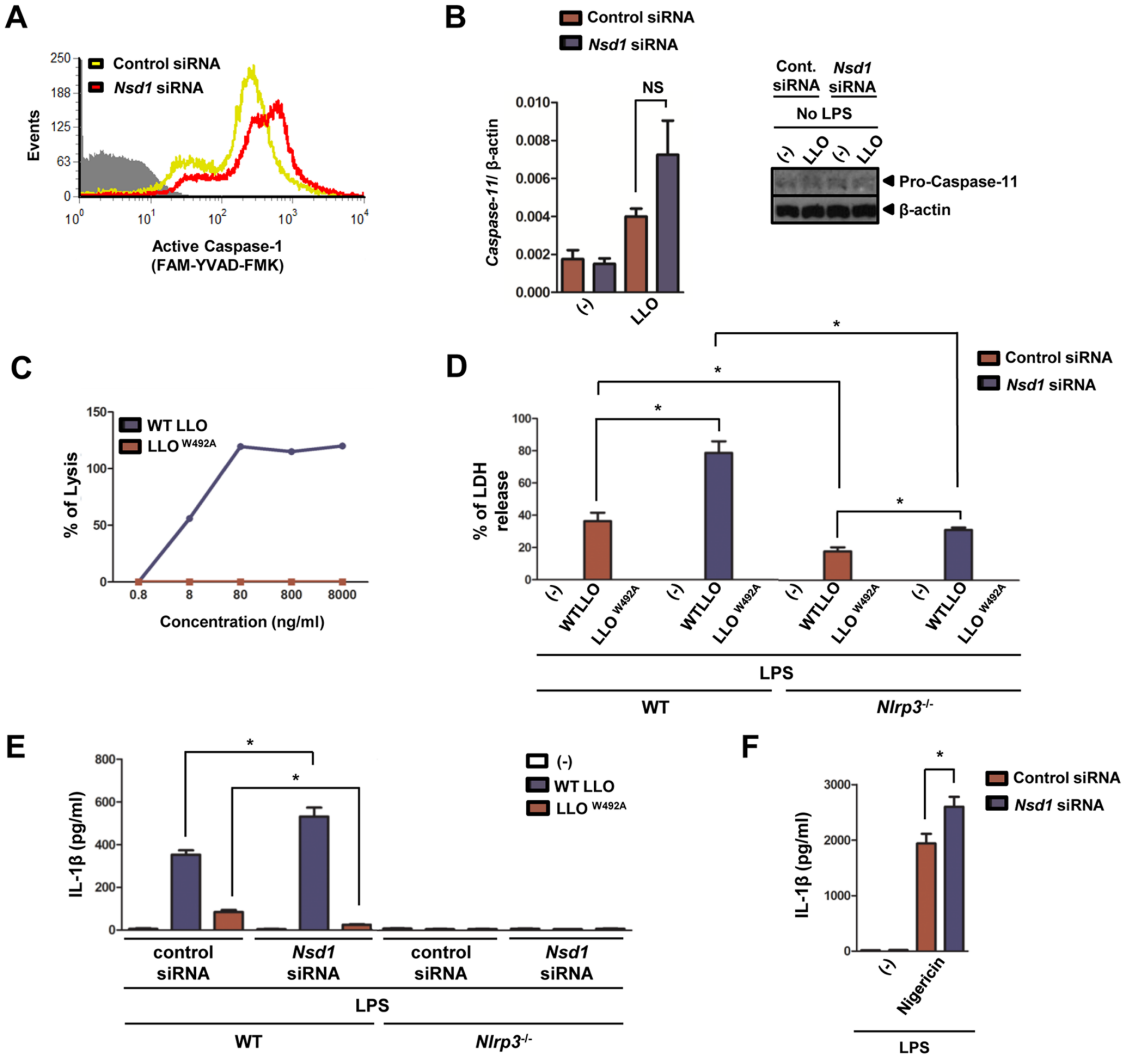
NSD1 inhibits LLO-mediated caspase-1 activation and requires functional LLO for the regulation of IL-1β secretion . 1×10^6^ BMDMs were primed with LPS (100 ng/ml) and treated with control silencer or *Nsd1* siRNA. Also, BMDMs were untreated or stimulated with LLO (500 ng/ml) for 30 minutes. (A) Caspase-1 activity in BMDMs (1×10^6^) transfected with *Nsd1* or control silencer siRNA was determined after LLO stimulation by flow cytometry using the fluorescent inhibitor probe FAM-YVAD-FMK (FLICA). Control cells are shown in gray, while cells treated with *Nsd1* or control siRNA are shown in red and yellow, respectively. Additionally, silenced 1×10^6^ BMDMs were primed with LPS (100 ng/ml) and were untreated or stimulated with LLO (8 µg/ml) for 1 hour. (B, *left*) RNA was collected and *caspase-11* transcription was analyzed by qPCR (n = 4). (B, *right*) Protein was also harvested and pro-caspase-11 translation was measured by immunoblot. β-actin was used to normalize for qPCR and determine equal loading for immunoblot. (C) Hemolytic assay was performed on sheep RBC using varying concentrations of recombinant WT LLO or LLO^W492A^. (D-E) 1×10^6^ WT BMDMs were primed with LPS (100 ng/ml) and treated with WT LLO or LLO^W492A^ (8 µg/ml) for 1 hour. (D) LDH assay was performed to measure cell death induction after treatment. (E) IL-1β levels secreted by BMDMs from WT (n = 3) and *Nlrp3* ^−/−^ (n = 4) mice were measured by ELISA. (F) IL-1β was measured by ELISA after nigericin (10 µM) stimulation (n = 4). Student's t test; (*) *P*<.05 compared to cells transfected with control siRNA or knockout. NS – not significant. Experiments were repeated at least twice.

We were prompted to investigate the involvement of NSD1 in the mediation of LLO-dependent NLRP3 inflammasome post-translational activity. An increase of active caspase-1 in macrophages treated with LLO relative to non-treated cells was detected (Fig. 7A). Furthermore, post-translational activation of caspase-1 was enhanced with *Nsd1* silencing in macrophages stimulated with LLO, as judged by flow cytometry (Fig. 7A). Recently, it was demonstrated that stimulation of macrophages with pathogenic bacteria, such as *Citrobacter rodentium* and *Vibrio cholera*, led to the activation of caspase-11 via TLR4-Toll/IL-1 receptor (TIR)-domain-containing adaptor inducing IFN-β (TRIF) signaling. This pathway then activates caspase-1 and enhances IL-1β secretion [57]–[59]. Arguing against the regulation of caspase-11 by NSD1, Kayagaki and collaborators have shown that caspase-1 activation in macrophages occurs independently of caspase-11 during LLO stimulation of macrophages [59]. Our data also supported this finding. The measurement of caspase-11 pre- or post-*Nsd1* reduction did not reveal differential transcription in the presence or absence of LLO (Fig. 7B, *left*). Correspondingly, differences in the caspase-11 protein were not observed despite stimulation with LLO (Fig. 7B, *right*).

To evaluate the importance of LLO pore formation on the NSD1 regulation of caspase-1, we compared caspase-1 activation during exposure to LLO and LLO^W492A^. This mutant lacks hemolytic activity due to a tryptophan to alanine substitution in the undecapeptide motif [60]. To assess the lytic activity of LLO and LLO^W492A^, hemolytic assays were performed (Fig. 7C). There was a dose-dependent increase in the percentage of lysis by LLO; a concentration as low as 8 ng/ml was able to promote membrane disruption. LLO^W492A^ demonstrated complete abrogation of red blood cell lysis. Additionally, LLO has been shown to trigger cell death [45]. To elucidate the role of NSD1 in the regulation of pyroptosis (caspase-1 mediated cell death), LDH assays were performed after macrophage stimulation with LLO and LLO^W492A^ (Fig. 7D). Stimulation of WT macrophages with LLO and LLO^W492A^ resulted in about 40% and 0% LDH release, respectively. After transfection of WT macrophages with *Nsd1* siRNA, supernatant LDH increased for WT LLO treated cells to 80%. However, cells treated with LLO^W492A^ still did not induce LDH release. This result implied that NSD1 plays a role in the inhibition of cell death during stimulation with functional LLO. In *Nlrp3* ^−/−^ macrophages, after control siRNA transfection, LLO released about 20% LDH, whereas the extracellular LDH level was about 30% for LLO-treated cells post-*Nsd1* silencing (Fig. 7D). In primed WT macrophages, LLO was able to induce IL-1β secretion after being transfected with control siRNA and levels of IL-1β increased after silencing with *Nsd1* siRNA (Fig. 7E). On the other hand, IL-1β secretion triggered by LLO^W492A^ was not mediated by NSD1 in the same manner as WT LLO. IL-1β release when *Nlrp3* ^−/−^ macrophages were stimulated with LLO and LLO^W492A^ was not observed (Fig. 7E). Finally, *Nsd1* silencing also affected IL-1β secretion during nigericin stimulation (Fig. 7F). Nigericin is a molecule that is similar to LLO in that it can lead to pore formation in the plasma membrane of macrophages [1]. Taken together, our findings suggest that alteration of the plasma membrane by pore-forming agents may be a factor for NSD1 mediated regulation of IL-1β secretion.

## Discussion

Discoveries made in plants have facilitated the understanding of innate immunity in mammals. Yet, several elegant studies are not translated into human health. This may be simply due to the lack of communication among scientists. Health scientists do not typically interact with plants researchers, and plant scientists are offered little incentives to interact with the biomedical research community. We took advantage of the NLR conservation in plants and mammals and discovered NSD1 as a possible regulator of the NLRP3 inflammasome. EDM2 was previously identified in a genetic screen for suppressors of the NLR gene RPP7 that provides resistance against the oomycete *H. parasitica* in plants [16]. EDM2 mutations phenocopied the RPP7 observation in *Arabidopsis*, and the defense mechanism was highly specific for *H. parasitica*.

Similar to the phenotype observed for EDM2 in *Arabidopsis*, we observed specificity for our NSD1 results in macrophages. *Nsd1* silencing affected the NLRP3 inflammasome when LLO stimulation of macrophages occurred. However, we did not notice any effect on alum, a NLRP3 stimulant via phagolysosomal instability, and *P. aeruginosa*, a NLRC4 stimulant [1]. These results may underscore the importance of pore formation for NSD1 regulation of the NLRP3 inflammasome. Pore-forming toxins are virulence proteins utilized by numerous bacteria in order to damage cell membranes [61]. Analysis of cholesterol-dependent cytolysins, perfringolysin O and intermedilysin, revealed that these pore-forming toxins have conserved structures and mechanisms of action [13]. Reiterating this hypothesis is the fact that *Nsd1* silencing also affects IL-1β secretion during stimulation of macrophages with the pore-forming agent nigericin. Future studies involving additional pore-forming toxins could reveal a broader application of NSD1 regulation on the NLRP3 inflammasome.

We tested the hypothesis that NSD1 could likely be a candidate for chromatin-based NLRP3 inflammasome regulation. However, after ChIP analysis, we were unable to identify a region with uniquely increased NSD1 enrichment. Effects of histone methylation were also not evident for inflammasome-related genes. This observation is not entirely surprising because a previous study observed that LLO-mediated histone modifications and inflammasome activation are independent pathways [45]. On the other hand, we focused our study on a commonly analyzed time point during inflammasome stimulation with LLO. Thus, although unlikely, our analysis may not entirely exclude the possibility that histone methylation by NSD1 could still be observed after macrophages are exposed to LLO, resulting in alteration of caspase-1 activation.

Our study focused on the interaction between NSD1 and caspase-1 because this inflammatory caspase is considered a canonical regulatory enzyme for IL-1β and IL-18 secretion. We do not exclude the possibility that NSD1 could target other caspases upstream of caspase-1, thereby, modulating IL-1β and IL-18 secretion. The measurement of caspase-11 pre- or post-*Nsd1* reduction did not reveal differential regulation in the presence or absence of LLO. A potential caspase of interest, caspase-7, has been shown to respond to *L. monocytogenes* and may act as a protective mechanism against membrane damage [36]. However, caspase-1 activation during *L. monocytogenes* infection is independent of caspase-7, as caspase-1-deficient mice did not show a defect in caspase-7 activation [36]. Another caspase known to form a non-canonical inflammasome is caspase-8. A relationship between caspase-8 and LLO has not been formally elucidated and, hence, we are not able to exclude any possible effects of NSD1 on caspase-8 activity during LLO stimulation of macrophages. Presently, caspase-8 has been known to associate with the AIM2-ASC complex during *Francisella tularensis* subspecies *novicida* stimulation of macrophages and dectin-1 recognition of fungi and mycobacteria [62], [63].

Clearly, additional studies encompassing NSD1 and the regulation of the canonical and non-canonical inflammasome components are necessary. However, our study potentially unveils a novel function for NSD1 during LLO stimulation of macrophages. Because the mechanism of action for many pore-forming toxins is evolutionarily conserved, our observation may also uncover a widely applicable regulatory innate immune mechanism in the mammalian host.
